# Effective Heat Transfer Pathways of Thermally Conductive Networks Formed by One-Dimensional Carbon Materials with Different Sizes

**DOI:** 10.3390/polym11101661

**Published:** 2019-10-11

**Authors:** Yun Seon Lee, Seung-Yong Lee, Keun Soo Kim, Suguru Noda, Sang Eun Shim, Cheol-Min Yang

**Affiliations:** 1 Institute of Advanced Composite Materials, Korea Institute of Science and Technology (KIST), 92 Chudong-ro, Wanju-gun, Jeonbuk 55324, Korea; t14225@kist.re.kr (Y.S.L.); seungyong@lginnotek.com (S.-Y.L.); 2 Department of Chemical Engineering, Inha University, 100 Inha-ro, Nam-gu, Incheon 22212, Korea; 3 Magok R&D campus, LG Innotek, 30 Magokjungang 10-ro, Gangseo-gu, Seoul 07796, Korea; 4 Department of Physics and Graphene Research Institute, Sejong University, 209 Neungdong-ro, Gwangjin-gu, Seoul 05006, Korea; kskim2676@sejong.ac.kr; 5 Department of Applied Chemistry, School of Advanced Science and Engineering, Waseda University, 3-4-1 Okubo, Shinjuku-ku, Tokyo 169-8555, Japan; noda@waseda.jp

**Keywords:** few-walled carbon nanotube, mesophase pitch-base carbon fiber, vacuum filtration, in-plane thermal conductivity, phonon scattering, laser flash technique

## Abstract

We investigated the heat transfer behavior of thermally conductive networks with one-dimensional carbon materials to design effective heat transfer pathways for hybrid filler systems of polymer matrix composites. Nano-sized few-walled carbon nanotubes (FWCNTs) and micro-sized mesophase pitch-based carbon fibers (MPCFs) were used as the thermally conductive materials. The bulk density and thermal conductivity of the FWCNT films increased proportionally with the ultrasonication time due to the enhanced dispersibility of the FWCNTs in an ethanol solvent. The ultrasonication-induced densification of the FWCNT films led to the effective formation of filler-to-filler connections, resulting in improved thermal conductivity. The thermal conductivity of the FWCNT-MPCF hybrid films was proportional to the MPCF content (maximum thermal conductivity at an MPCF content of 60 wt %), indicating the synergistic effect on the thermal conductivity enhancement. Moreover, the MPCF-to-MPCF heat transfer pathways in the FWCNT-MPCF hybrid films were the most effective in achieving high thermal conductivity due to the smaller interfacial area and shorter heat transfer pathway of the MPCFs. The FWCNTs could act as thermal bridges between neighboring MPCFs for effective heat transfer. Furthermore, the incorporation of Ag nanoparticles of approximately 300 nm into the FWCNT-MPCF hybrid film dramatically enhanced the thermal conductivity, which was closely related to a decreased thermal interfacial resistance at the intersection points between the materials. Epoxy-based composites loaded with the FWCNTs, MPCFs, FWCNT-MPCF hybrids, and FWCNT-MPCF-Ag hybrid fillers were also fabricated. A similar trend in thermal conductivity was observed in the polymer matrix composite with carbon-based hybrid films.

## 1. Introduction

In recent years, as electronic devices have been downsized, integrated, and functionalized, the total amount of heat generation during their operation has rapidly increased. Therefore, it is necessary to dissipate the heat generated from such devices effectively to enhance their performance, lifetime, and reliability, which has resulted in a rapid increase in the market demand for high-performance heat-dissipation materials [1,2,3,4,5,6]. Thermally conductive materials with high thermal conductivity have frequently been used in heat-dissipation components with two- or three-dimensional forms such as the heat spreader, thermal interface material, and heat sink [1]. The thermally conductive materials are used alone or in composites with various matrix or binder materials [1,2,5]. In particular, polymer composite materials have been actively studied as alternative heat-dissipation materials [1,3,5]. Despite their numerous advantages including low cost, light weight, and excellent processibility compared to other thermally conductive materials such as metals and ceramics, they exhibit low thermal conductivity, which is a serious drawback for extensive applications as heat-dissipation materials [1,5]. In general, these polymers exhibit an extremely low thermal conductivity of less than 0.5 W/mK. Therefore, various researches have been carried out to develop polymers with high thermal conductivity [1,3,5]. The most common approaches involve combining the polymers with thermally conductive fillers [1,3,5]. The thermal conductivity of polymer composites is strongly influenced by the intrinsic thermal conductivity, loading amount, shape, and size of the fillers. Various carbon-based fillers such as graphite, carbon fiber (CF), carbon nanotube (CNT), and graphene have been used as thermally conductive fillers for polymer composites in applications where electrical insulation is not required [1,3,5,7,8,9]. However, the optimal heat transfer pathways in single or hybrid fillers with high thermal conductivity in composite systems remain unclear.

In particular, CNTs and CFs are representative one-dimensional (1D) fillers with high intrinsic thermal conductivity along the longitudinal direction. CNTs have an extremely high intrinsic thermal conductivity (approximately 3000 W/mK) due to their long phonon free path in a 1D structure composed of sp^2^ carbons, which have nanometer-scaled diameters and sub-millimeter-scaled lengths [10,11]. Therefore, CNT-polymer composites offer great potential for applications in thermal management fields, owing to the expectation of high thermal conductivity. However, contrary to this expectation, the thermal conductivity of CNT-polymer composites is much lower than that of an individual CNT [5]. This is because CNTs have a large number of structural defects, and in particular, the thermal conductivity of the CNT network is highly dominated by the thermal contact resistance at junctions formed between the CNTs [12]. Therefore, the thermal conductivity may be increased by increasing the intrinsic crystallinity of the CNTs and reducing the thermal contact resistance between the CNTs as far as possible [12]. As CNTs are interconnected by van der Waals forces, they can be formed into a paper-like film without any other support (so-called buckypaper) [13,14,15]. Mesophase pitch-based carbon fibers (MPCFs) with a 1D structure and micrometer-scaled diameter also exhibit high thermal conductivity (approximately 1000 W/mK), originating from their highly crystalline graphitic structure [16]. Therefore, MPCFs have attracted significant attention as thermally conductive reinforcements of polymer composites for high heat dissipation [17]. CNTs and MPCFs are expected to achieve a low percolation threshold in polymer-based composites given their high aspect ratios.

In this study, we investigated the heat transfer behavior of thermally conductive networks consisting of nano-sized and/or micro-sized carbon materials with a 1D structure to comprehend the effects of hybrid filler systems on thermal conductivity in various composites. We used few-walled CNTs (FWCNTs) with a long length (approximately 400 μm) and high aspect ratio (approximately 50,000) as the thermally conductive material with nano-sizes and 1D shapes, which was expected to be more efficient for heat transfer [18]. We also used the MPCFs as thermally conductive materials with micro-sizes and 1D shapes. Free-standing FWCNT and FWCNT-MPCF hybrid films were fabricated using the ultrasonication-assisted vacuum filtration method. Moreover, Ag nanoparticles were incorporated into the FWCNT-MPCF hybrid films. We also investigated the thermal conductivity of epoxy-based composites loaded with the FWCNTs, MPCFs, FWCNT-MPCF hybrids, and FWCNT-MPCF-Ag hybrid fillers.

## 2. Materials and Methods

### 2.1. Thermally Conductive Filler Materials

The free-standing flexible carbon-based films used in this study were prepared using sub-millimeter-long FWCNTs (6 to 10 nm diameter, 400 μm length, triple-walled on average). Detailed experimental information on the FWCNT synthesis was presented in our previous reports [18]; accordingly, only brief explanations are provided here. The FWCNTs were grown by the novel semi-continuous fluidized-bed chemical vapor deposition process in a quartz-tube thermal furnace with an Fe/Al_2_O_3_ catalyst deposited onto Al_2_O_3_ beads by feeding metalorganic vapors of aluminum-isopropoxide (95%, Wako, Osaka, Japan) and ferrocene (98%, Wako, Osaka, Japan) sequentially, using a carrier gas of O_2_/Ar balance [18]. Subsequently, the catalyst was reduced by flowing an H_2_/H_2_O/Ar balance for 10 min, following which the FWCNTs were synthesized on the Al_2_O_3_ beads with a C_2_H_2_/H_2_/H_2_O/Ar atmosphere for 10 min [19]. The MPCFs (Dialead, K223HE, 11 μm diameter, 200 μm and 6 mm length) were purchased from Mitsubishi Plastic Inc. (Tokyo, Japan). The organic Ag (5% organic Ag methanol dispersion, amine-based silver chelate mixture) was purchased from Ditto Technology Co. Ltd (Gunpo-si, Gyeonggi-do, Korea).

### 2.2. Preparation of Flexible Carbon-Based Films

**Figure 1:**
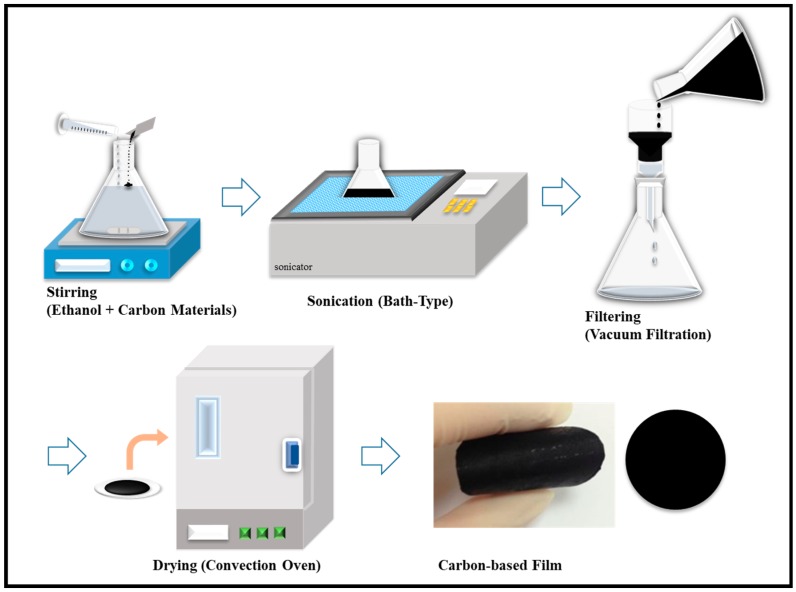
Schematic of the fabrication process of carbon-based films.

To prepare the free-standing flexible carbon-based films by means of a standard vacuum filtration technique, the carbon materials (FWCNTs and MPCFs, 50 mg) and Ag (0 to 25 mg) were first dispersed in ethanol (500 mL) without any pretreatment and surfactant, using bath sonication (40 kHz, 300 W) for different ultrasonication times of 0.5, 2, 4, 8, and 12 h. The carbon suspensions were vacuum-filtrated using a polytetrafluoroethylene (PTFE) membrane filter (pore diameter: 10 μm, Millipore, Darmstadt, Germany), and then washed with distilled water several times to obtain pure carbon-based films. As the solvent fell through the pores, the FWCNTs were stacked onto the PTFE membrane filter surface, forming a randomly entangled network. After the suspension was completely dried out on a filter in a vacuum oven at 100 °C overnight, the carbon-based films were carefully peeled off from the PTFE membrane filter. The diameter of the obtained carbon-based films was approximately 35 mm, as illustrated in Figure 1. To examine the influence of defects on the thermal conductivity, the pristine carbon-based films were annealed at 1000 °C for 3 h in an Ar atmosphere (approximately 0.4 torr) prior to the in-plane thermal conductivity measurements. To eliminate organic materials from the Ag nanoparticles, FWCNT-MPCF-Ag hybrid films were annealed at 300 °C for 1 h in an air atmosphere. The detailed material properties of the carbon-based films were characterized using high-resolution scanning electron microscopy (HR-SEM, Nova Nano SEM 450, FEI, Hillsboro, OR, USA) and micro-Raman spectroscopy with an excitation laser wavelength of 514 nm (InVia, Renishaw, Wotton-under-Edge, Gloucestershire, UK). Thermogravimetric analysis (TGA, Q50, TA Instruments, Inc., New Castle, DE, USA) was carried out under an air atmosphere up to 350 °C (with a heating rate of 10 °C min^−1^).

### 2.3. Preparation of Thermally Conductive Epoxy-Based Composites

The epoxy-based composites containing only FWCNTs, only MPCFs (200 μm length), FWCNT-MPCF hybrids (MPCF length of 200 μm or 6 mm; FWCNT:MPCF weight ratio of 40:60), and MWCNT-MPCF-Ag hybrid fillers (MPCF length of 6 mm; FWCNT:MPCF weight ratio of 40:60; Ag content: 5 phr) were prepared. The total loading amounts of MWCNTs and MPCFs in the epoxy-based composites were adjusted to 10 wt %. The epoxy resin (DGEBA, YD-128) and curing agent (Jeffamin D-230) were purchased from Kukdo Chemical Co. Ltd. (Seoul, Korea). The composition of the epoxy and curing agent was fixed at a weight ratio of 7:3 for this study. The epoxy resin was diluted with ethanol before adding the fillers; this allowed for a uniform dispersion of thermally conductive fillers within the epoxy resin. The mixture of epoxy resin, ethanol, and thermally conductive fillers underwent mechanical stirring for 0.5 h with subsequent bath sonication (40 kHz, 300 W) for 0.5 h. The ethanol in the mixture was evaporated in a hot plate at 75 °C for 1 h. Subsequently, the curing agent was added to the mixture, which was mechanically stirred for 0.5 h. The mixture was cured at 100 °C for 1 h in a mold that was 20 mm wide, 40 mm long, and 250 μm thick.

### 2.4. In-Plane Thermal Conductivity Measurements of Flexible Carbon-Based Films and Epoxy-Based Composites

The in-plane thermal conductivity of the carbon-based films was measured by means of a “modified laser flash” technique in the LFA 447 Nanoflash instrument (Xenon flash lamp, Netzsch Instruments, Inc., Selb, Germany) at room temperature. Conventionally, the laser flash technique has been used extensively to investigate the out-of-plane thermal conductivity of various films and bulk materials [20,21]. To measure the in-plane heat dissipation properties of the carbon-based films, the cylindrical disc-shaped specimens of 25.4 mm in diameter were positioned in a special sample holder equipped with a temperature sensor, allowing for measurement of the temperature changes along the in-plane direction, as illustrated in Figure S1 of the Supplementary Material. The specimen front surface was illuminated by the flash of a Xenon lamp (wavelength (λ) = 150–2000 nm), following which the temperature changes in the specimen rear surface were monitored by a cryogenically cooled InSb IR detector. The measured temperature as a function of time at the specimen’s rear surface could be ensured as the distance of the heat traveled along the in-plane direction was fairly long when compared to that of the heat moved along the out-of-plane direction. The thermal diffusivities (*α*) of the carbon films can be calculated using the following equation [22]:

$$\alpha =\frac{0.1388L^{2}}{t_{50\%}}$$

where *α* and *L* are the thermal diffusivity and thickness of the carbon films, respectively, and *t*_50%_ is the time for the rear surface to reach 50% of the maximum temperature value, as described in Figure S1 of the Supplementary Material. The thermal diffusivity measurements of the specimens were carried out three times, and the experimental uncertainty (Netzsch Instruments, Inc., Bavarian, Germany) was less than ±3%. The specific heat (*C_p_*) of the carbon-based films was estimated based on that of graphite, namely 704 J/kg∙K at room temperature, as the temperature-dependent specific heat of the CNTs converges with that of graphite at temperatures exceeding approximately 100 K [23]. The bulk density (*ρ*) of the carbon-based films was determined by measuring the volume (thickness and diameter) and mass. The density (*ρ*) of the epoxy-based composites was measured using the Archimedes’ principle. The specific heat (*C_p_*) of the epoxy-based composites was measured using a differential scanning calorimeter (DSC, Q20, TA Instruments, Inc., New Castle, PA, USA) at room temperature. The thermal diffusivity (*α*) of the epoxy-based composites was measured using a Laser PIT (Ulvac Riko, Inc., Yokohama, Japan) at room temperature. The in-plane thermal conductivity (*κ*) of the carbon-based films and epoxy-based composites is expressed by Equation (2), with the obtained thermal diffusivity (*α*), specific heat (*C_p_*), and bulk density (*ρ*) as follows:

(2) $$\kappa =\alpha C_{p}\rho$$

## 3. Results and Discussion

### 3.1. Surface Morphology and Thermal Conductivity of Few-Walled Carbon Nanotube (FWCNT) Films

**Figure 2:**
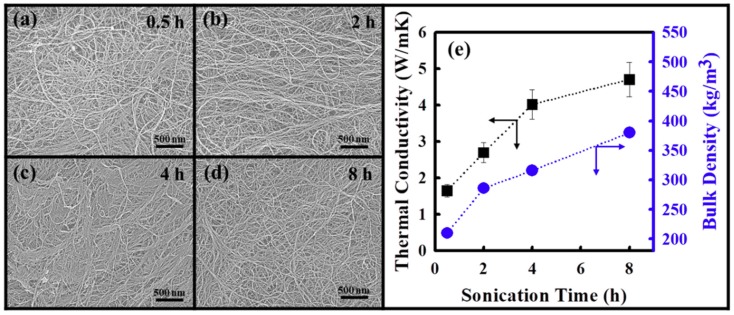
High-resolution scanning electron microscopy (HR-SEM) images of few-walled carbon nanotube (FWCNT) films prepared following ultrasonication for different times: (**a**) 0.5 h, (**b**) 2 h, (**c**) 4 h, and (**d**) 8 h; (**e**) thermal conductivity (black quadrangles and arrows) and film bulk density (blue circles and arrows) of FWCNT films with different sonication times.

In general, free-standing CNT films derived from single-walled CNTs can be easily fabricated by the vacuum filtration process due to their tendency to form a bundled assembly structure [24,25,26]. However, the film formation of multi-walled CNT (MWCNT) structures is known to be difficult, because of their entangled assembly structure [27,28]. Free-standing FWCNT film can be prepared by the vacuum filtration process following ultrasonication in only 0.5 h. We assumed that the FWCNTs should be easily disentangled by applying an external physical force such as ultrasonication, and the filtrated FWCNTs should be transformed into a more developed bundle structure, resulting in the formation of a free-standing FWCNT film. As indicated in the HR-SEM images of the FWCNT films in Figure 2a–d, the FWCNT films exhibited the network structure of continuous FWCNTs, which was the result of the self-assembly of long CNTs by van der Waals forces during ultrasonication-assisted vacuum filtration [29]. Moreover, it was confirmed that the FWCNT films were densified as the ultrasonication treatment time increased. We assumed that the formation of the free-standing CNT film should be strongly dependent on the degree of CNT entanglement. Therefore, FWCNTs with a low degree of entanglement are easily disentangled by ultrasonication treatment, and the disentangled FWCNTs are rearranged and bundled on the membrane filter during vacuum filtration, which can be promoted by a longer ultrasonication treatment time. This indicates the excellent film formation ability of FWCNTs.

The in-plane thermal conductivity and bulk density of the FWCNT films according to the ultrasonication treatment time are illustrated in Figure 2e. It can be observed that the thermal conductivity and bulk density of the FWCNT films increased from 1.65 to 4.71 W/mK (by 185%) and from 210 to 360 kg/m^3^ (by 71%), respectively, as the ultrasonication treatment time increased up to 8 h. This result indicates that the dispersibility of the FWCNTs increases with an increased ultrasonication treatment time, and consequently, the bulk density of the FWCNT films increases due to a decrease in the voids of the FWCNT films originating from the large inter-bundle space. The thermal diffusivity of the FWCNT films increases with a decrease in the phonon scattering at the junctions between the FWCNTs. As a result, the thermal conductivity of the FWCNT films increases with an increase in their thermal diffusivity and bulk density. This result clearly indicates that the density of the FWCNT films plays a dominant role in heat transfer in FWCNT networks. Therefore, this bulk density of the carbon-based films can be associated with the loading amount of thermally conductive carbon-based fillers in composite systems, which plays a major role in determining the thermal conductivity.

### 3.2. Surface Morphology and Thermal Conductivity of Few-Walled Carbon Nanotube (FWCNT)- Mesophase Pitch-Based Carbon Fiber (MPCF) Hybrid Films

**Figure 3:**
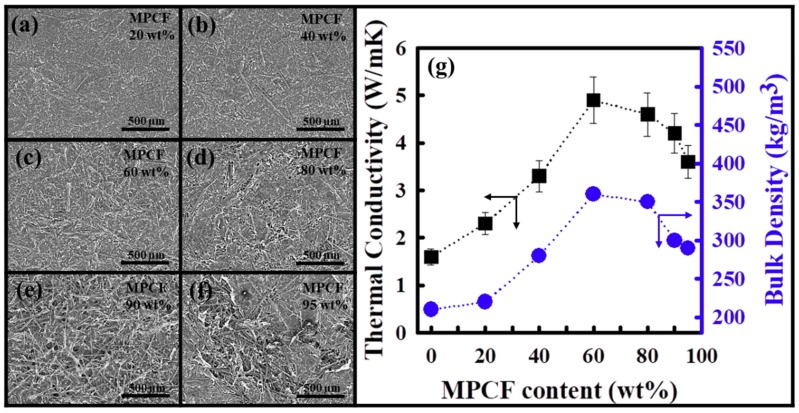
HR-SEM images of the FWCNT-MPCF hybrid films with different mesophase pitch-based carbon fiber (MPCF) (length: 200 μm) contents: (**a**) 20 wt %, (**b**) 40 wt %, (**c**) 60 wt %, (**d**) 80 wt %, (**e**) 90 wt %, and (**f**) 95 wt %; (**g**) thermal conductivity (black quadrangles and arrows) and film bulk density (blue circles and arrows) of the FWCNT-MPCF hybrid films with different MPCF (length: 200 μm) contents. These FWCNT-MPCF films were prepared by vacuum filtration following ultrasonication for 0.5 h.

To elucidate the size effect of the 1D-shaped carbon materials on the thermal conductivity of carbon films, FWCNT-MPCF hybrid films were fabricated using the same method as that of the FWCNT films with an ultrasonication time of 0.5 h (milled MPCF length of 200 μm). The surface morphology of the FWCNT-MPCF hybrid films was analyzed by means of HR-SEM, as illustrated in Figure 3a–f. When the MPCF contents were less than 60 wt %, the surface morphology of the films was smooth and the MPCFs were uniformly distributed among the FWCNTs. However, when the MPCF contents were more than 80 wt %, numerous large voids could be observed between the MPCFs due to the small quantity of FWCNTs (Figure S2 of the Supplementary Material). This is because MPCFs with large diameters are relatively stiff, while FWCNTs with very small diameters are more flexible. This phenomenon appears to occur when the MPCF content reaches a critical amount (80 wt % in our case). The thermal conductivity and bulk density of the FWCNT-MPCF hybrid films are presented in Figure 3g. For MPCF contents of less than 60 wt %, the thermal conductivity values of the FWCNT-MPCF hybrid films were linearly proportional to the MPCF content, indicating an obvious synergistic effect on the thermal conductivity by means of the hybridization of two fillers. The FWCNT-MPCF hybrid film with an MPCF content of 60 wt % had the highest thermal conductivity value of 4.91 W/mK (an approximately 200% increase compared to the FWCNT film of 1.65 W/mK). This is because the bulk density of the FWCNT-MPCF hybrid film increased with an increasing MPCF content, and thereby, short and direct pathways for heat transfer were effectively formed. However, for MPCF contents of more than 80 wt %, the thermal conductivity of the FWCNT-MPCF hybrid films decreased, which could be attributed to the increase in the film voids, and thereby, the decrease in the film bulk density, as confirmed in the HR-SEM images. Therefore, it is necessary to form an optimal network structure with both high film bulk density and short heat transfer pathways to obtain a hybrid film with high thermal conductivity.

### 3.3. Effect of Thermal Annealing on Thermal Conductivity of FWCNT-MPCF Hybrid Films

**Figure 4:**
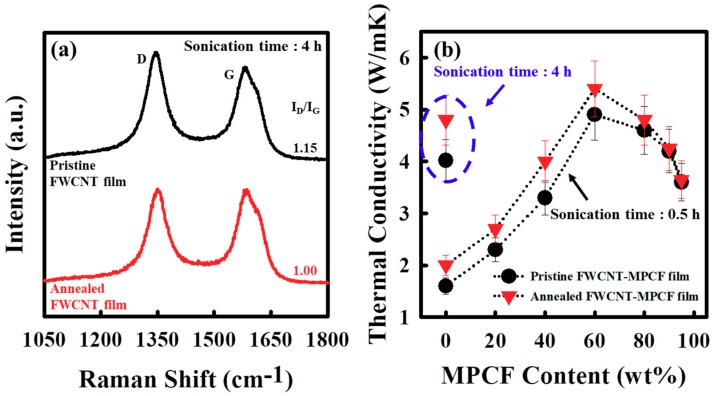
(**a**) Raman spectra of the pristine and annealed (at 1000 °C) FWCNT films (ultrasonication for 4 h) (The *D*-band (approximately 1350 cm^−1^) and G-band (approximately 1580 cm^−1^) are related to defect-induced structural disorders and *sp^2^* carbons, respectively.); (**b**) thermal conductivity of FWCNT-MPCF hybrid films with different MPCF content (length: 200 μm; ultrasonication for 0.5 or 4 h) before and after thermal annealing (at 1000 °C).

We examined the thermal annealing effect of the FWCNT and FWCNT-MPCF hybrid films on their structural and thermal characteristics. The structural properties of the pristine and annealed FWCNT films were analyzed by means of Raman spectroscopy. Raman spectroscopy has been used extensively as a powerful tool for characterizing carbon materials in terms of their structural properties and qualities [30,31,32]. One of the most informative parameters in the Raman characterization of carbon materials is the ratio between the integrated intensities of the *D*- and *G*-bands (*I_D_*/*I_G_*) [33]. The *D*-band of approximately 1350 cm^−1^ is closely related to defect-induced structural disorders. Carbon materials also exhibit the well-known *G*-band characteristic of *sp*^2^ carbons at approximately 1580 cm^−1^. Thus, the *I_D_*/*I_G_* ratio is effective in examining the crystallinity and structural disorder of carbon-based films. Figure 4a illustrates the Raman spectra (excited at 514 nm) for the pristine and thermally annealed FWCNT films (with a sonication time of 4 h). The Raman spectrum of the pristine FWCNTs exhibits similar features to that of general MWCNTs, originating from the numerous pentagonal and heptagonal defects on the FWCNTs. The *I_D_*/*I_G_* ratio of the thermally annealed FWCNT film (*I_D_*/*I_G_* = 1.0) was lower than that of the pristine FWCNT film (*I_D_*/*I_G_* = 1.15), indicating a decrease in the defect concentration of the FWCNTs following thermal annealing at a temperature of 1000 °C. Moreover, the thermal conductivity of the FWCNT film (with a sonication time of 4 h) increased from 4.02 to 4.80 W/mK due to the enhanced crystallinity by means of the decrease in the defect concentration of the FWCNTs (Figure 4b). This result is strongly consistent with the Raman spectroscopy data. The thermal conductivity values of the FWCNT-MPCF hybrid films (with a sonication time of 0.5 h) following thermal annealing at 1000 °C are also presented in Figure 4b. When the MPCF content was less than 80 wt %, the thermal conductivity values of the thermally annealed films were higher than those of the pristine films. However, the thermal annealing for the FWCNT-MPCF hybrid films with a MPCF content of more than 90 wt % resulted in almost no change in the thermal conductivity values. The main reason for this will be described below.

**Figure 5:**
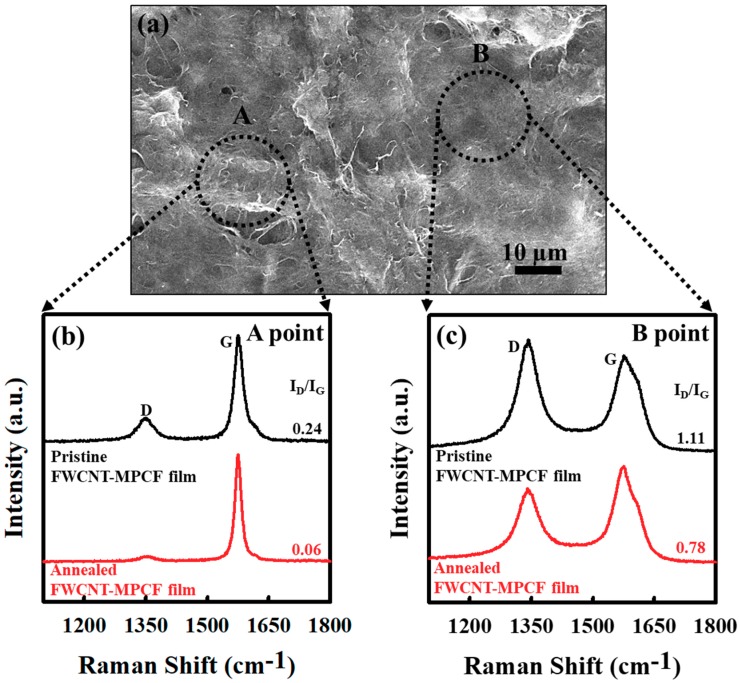
(**a**) HR-SEM images of FWCNT-MPCF hybrid film with MPCF (length: 200 μm) content of 60 wt % (ultrasonication for 0.5 h); (**b**,**c**) Raman spectra at points “A” and “B” of FWCNT-MPCF hybrid film with MPCF content of 60 wt % (ultrasonication for 0.5 h) before and after thermal annealing at 1000 °C. The *D*-band (approximately 1350 cm^−1^) and G-band (approximately 1580 cm^−1^) are related to defect-induced structural disorders and *sp^2^* carbons, respectively.

To clarify the relationship between the changes in thermal conductivity and crystallinity by the annealing effect, two different positions in the FWCNT-MPCF hybrid film were analyzed using Raman spectroscopy. Figure 5 presents the HR-SEM image and Raman spectra of the FWCNT-MPCF hybrid film with 60 wt % MPCFs (with a sonication time of 0.5 h). Point “A” is a network structure with both the MPCF and FWCNTs, while point “B” is a network structure with only FWCNTs. The Raman spectrum at point “A” of the pristine FWCNT-MPCF hybrid film indicates a graphitic structure with high crystallinity, which is a typical characteristic of carbon fiber graphitized at a temperature of approximately 3000 °C (inert atmosphere) [34]. However, the Raman spectrum at point “B” of the pristine FWCNT-MPCF hybrid film exhibits characteristics of the FWCNTs. The *D*-band at point “A” was much smaller than that at point “B”, indicating the developed graphitic structure of sp^2^ carbons of the MPCF. The *I_D_*/*I_G_* values of the thermally annealed FWCNT-MPCF hybrid film were lower than those of the pristine film at both points “A” and “B”. We assumed that the MPCFs with high crystallinity had a much smaller annealing effect on the thermal conductivity when compared to the FWCNTs with high-concentration defects. For this reason, the FWCNT-MPCF hybrid films with MPCF contents of more than 90 wt % were almost unchanged in terms of thermal conductivity by thermal annealing, as illustrated in Figure 4b.

### 3.4. Effect of the MPCF Length on Thermal Conductivity of FWCNT-MPCF Hybrid Films

**Figure 6:**
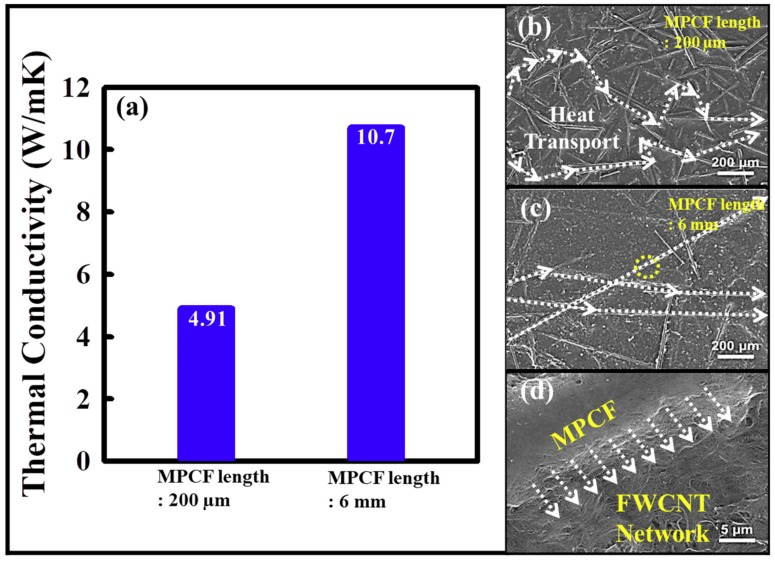
(**a**) Thermal conductivity and (**b**–**d**) HR-SEM images of FWCNT-MPCF hybrid films with different MPCF lengths of 200 μm and 6 mm (MPCF content: 60 wt %; ultrasonication for 0.5 h). (**d**) is high-resolution HR-SEM image of yellow circle in (**c**). The white arrows in (**b**–**d**) indicate the heat transfer pathways in carbon-based hybrid films.

To examine the effect of the MPCF length on the thermal conductivity, FWCNT-MPCF hybrid films were prepared using short-milled MPCFs (200 μm) and long-chopped MPCFs (6 mm) with a 60 wt %, respectively (with an ultrasonication time of 0.5 h). In this case, we selected an ultrasonication time of 0.5 h because of the thermal conductivity comparison in terms of only different MPCF lengths, which was the minimum time required for the formation of the free-standing film in our study. Figure 6 presents the thermal conductivity and HR-SEM images of the FWCNT-MPCF hybrid films prepared with MPCFs of different lengths. The thermal conductivity of the long MPCF film was 10.7 W/mK, which was 118% higher than that of the short MPCF film (4.91 W/mK), as indicated in Figure 6a. As demonstrated in the HR-SEM images shown in Figure 6b, the lower thermal conductivity of the short MPCF film was associated with increased phonon scattering at numerous inter-fiber junctions during the heat transfer. However, the heat transfer pathways of the long MPCF film were shorter than those of the short MPCF film, resulting in reduced resistance in the inter-fiber junctions of the heat conduction network of the FWCNT-MPCF hybrid film (Figure 6c). Based on these results, the main heat transfer pathways were as follows: (1) MPCF to MPCF, (2) MPCF to FWCNT, (3) FWCNT to MPCF, and (4) FWCNT to FWCNT. Among these, the short heat transfer pathway from MPCF to MPCF was the most important pathway for high thermal conductivity, which had the least phonon scattering. Therefore, as the MPCF content in the FWCNT-MPCF hybrid films increased, the heat transfer pathways between the MPCFs became shorter, and thereby, the thermal conductivity increased. Moreover, the FWCNTs in the FWCNT-MPCF hybrid films could act as thermal bridges between neighboring MPCFs for the formation of effective heat transfer pathways (Figure 6d). Therefore, if the amount of nanometer-sized FWCNTs is excessively small, the heat transfer pathways between the MPCFs and FWCNTs are cut off, and the thermal conductivity decreases.

### 3.5. Effect of Incorporation of Ag Nanoparticles on the Thermal Conductivity of FWCNT-MPCF Hybrid Films

**Figure 7:**
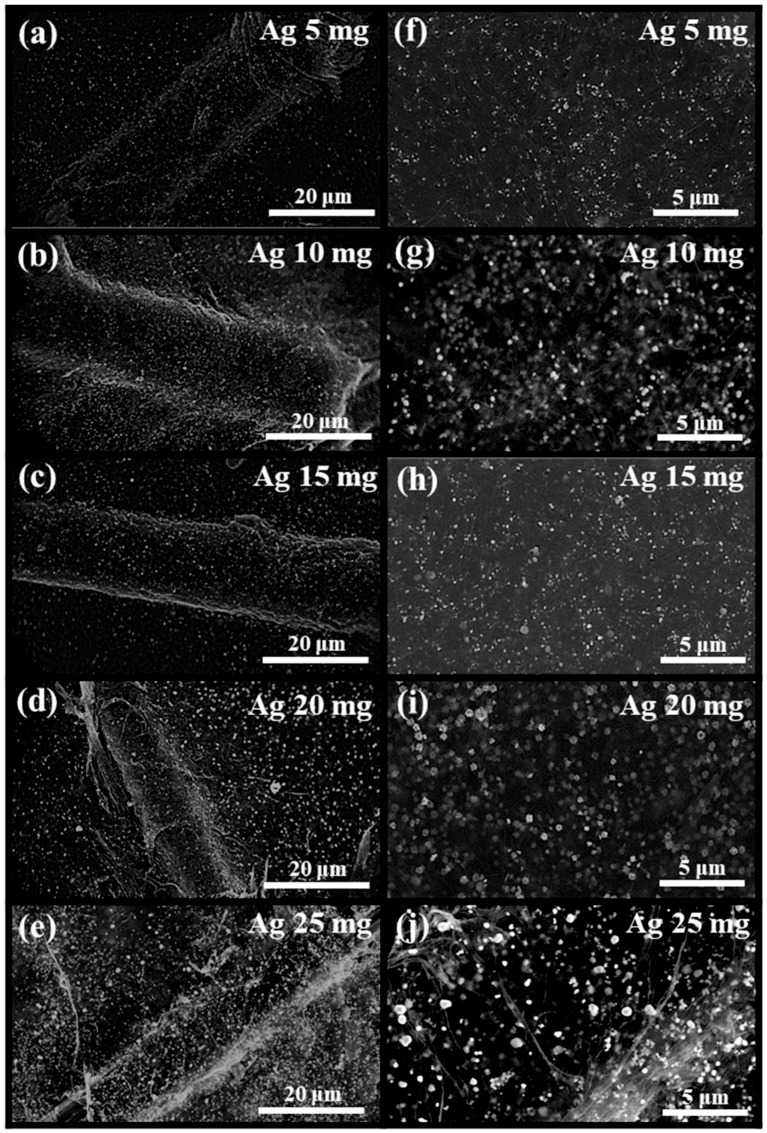
HR-SEM images of the FWCNT-MPCF-Ag hybrid films incorporated with different Ag contents of: (**a**,**f**) 5 mg (9.1 wt %), (**b**,**g**) 10 mg (16.6 wt %), (**c**,**h**) 15 mg (23.0 wt %), (**d**,**i**) 20 mg (28.6 wt %), and (**e**,**j**) 25 mg (33.3 wt %) (MPCF length: 6 mm; MPCF content: 30 mg; FWCNT content: 20 mg; ultrasonication for 0.5 h). (**a**–**e**) are low-magnification images, while (**f**–**j**) are high-magnification images.

**Figure 8:**
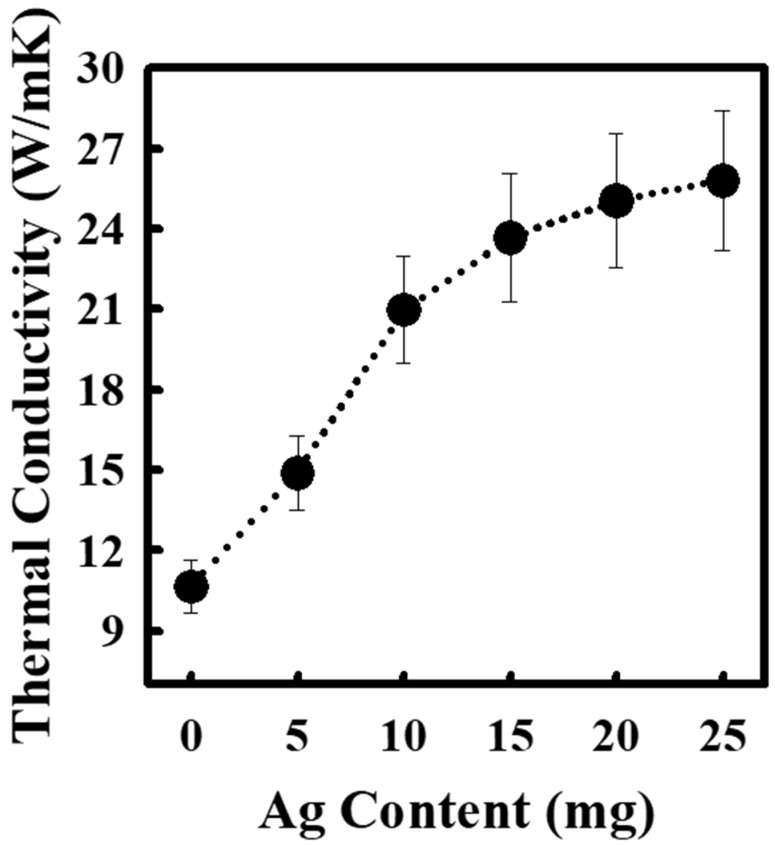
Thermal conductivity of FWCNT-MPCF-Ag hybrid films incorporated with different Ag content (MPCF length: 6 mm; MPCF content: 30 mg; FWCNT content: 20 mg; ultrasonication for 0.5 h).

According to the previous results, the thermal conductivities of carbon-based films are closely related to the intersection points in the random network of thermally conductive FWCNTs and MPCFs. Therefore, to reduce the phonon scattering at the intersection points between the thermally conductive carbon-based materials, Ag nanoparticles were incorporated into the FWCNT-MPCF hybrid films (MPCF length: 6 mm). The Ag nanoparticles used were in the form of organic Ag with amine groups to increase dispersibility in the solvent. Following the formation of free-standing FWCNT-MPCF hybrid films incorporated with organic Ag nanoparticles, the amine groups were removed by heat treatment at 300 °C (air atmosphere). The TGA result indicates that the weight loss of the Ag nanoparticles at 300 °C was 4.3 wt %, as illustrated in Figure S3 of the Supplementary Material. The surface morphology of the FWCNT-MPCF-Ag hybrid films was observed by HR-SEM, the images of which are presented in Figure 7a–j. It can be confirmed that the Ag nanoparticles were uniformly distributed in the FWCNT-MPCF-Ag hybrid film. The average Ag nanoparticle size was approximately 300 nm, which was measured using the HR-SEM images (Figure S4 of the Supplementary Material). According to the thermal conductivity measurement, the thermal conductivity of the FWCNT-MPCF-Ag hybrid films increased dramatically from 10.7 to 25.8 W/mK by adding Ag nanoparticles of 25 mg (33.3 wt %), and proportionally increased with an increase in the contents of the incorporated Ag nanoparticles in the FWCNT-MPCF hybrid films, as illustrated in Figure 8. This result clearly demonstrates that the Ag nanoparticles could act as a thermal bridge for the heat transfer between the carbon materials, thereby reducing the phonon scattering and increasing the thermal conductivity of the carbon-based hybrid films.

### 3.6. Thermal Conductivity of Epoxy-Based Composites

**Figure 9:**
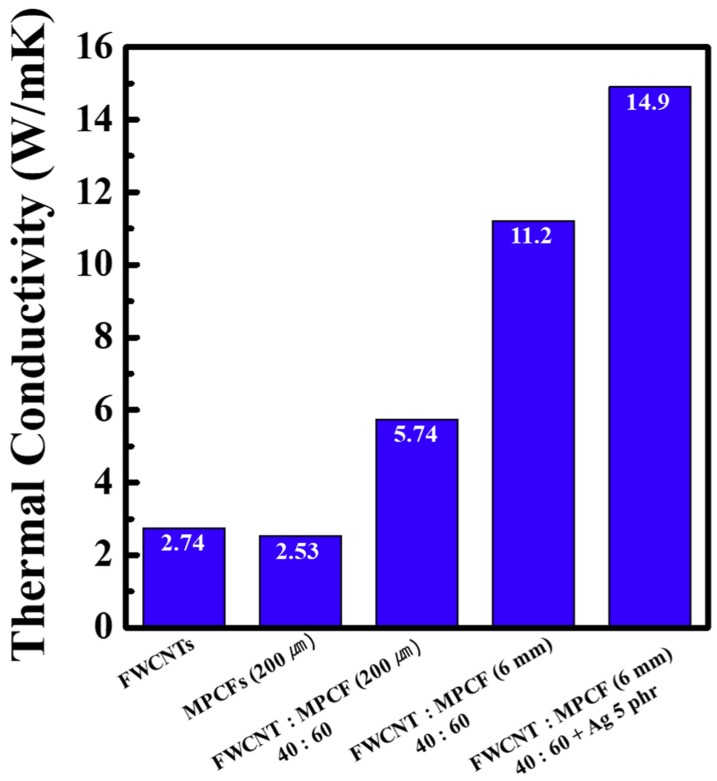
Thermal conductivity of epoxy-based composites containing only FWCNTs, only MPCFs (200 μm length), FWCNT-MPCF hybrids (MPCF length of 200 μm or 6 mm; FWCNT:MPCF weight ratio of 40:60), and MWCNT-MPCF-Ag hybrids (MPCF length of 6 mm; FWCNT:MPCF weight ratio of 40:60; Ag content: 5 phr). The total loading amounts of MWCNTs and MPCFs in the epoxy-based composites were adjusted to 10 wt %.

We investigated the thermal conductivity of epoxy-based composites loaded with hybrid fillers. This was aimed at elucidating the effects of thermally conductive filler networks on thermal transport properties in polymer matrix composites. Figure 9 presents the thermal conductivity of epoxy-based composites containing only FWCNTs, only MPCFs, FWCNT-MPCF hybrids with different MPCF lengths, and FWCNT-MPCF-Ag hybrid fillers. The FWCNT/epoxy and MPCF/epoxy (MPCF length of 200 μm) composites at a filler loading of 10 wt % exhibited relatively low thermal conductivity values of 2.74 and 2.53 W/mK, respectively. The epoxy-based composite consisted of a FWCNT-MPCF hybrid filler (MPCF length of 200 μm; FWCNT:MPCF weight ratio of 40:60) with a total filler loading of 10 wt %. This composite dramatically increased the thermal conductivity value of 5.74 W/mK compared to the FWCNT/epoxy and MPCF/epoxy composites; this indicates the influence of a synergistic effect on the thermal conductivity, which was caused by the hybridization of two fillers. The thermal conductivity of the epoxy-based composite with the FWCNT-MPCF hybrid filler using the long MPCF filler (MPCF length of 6 mm; FWCNT: MPCF weight ratio of 40:60) increased by 95% when compared to that of the composite using the short MPCFs (from 5.74 to 11.2 W/mK). These thermal conductivity results were in good agreement with those of the free-standing films. The thermal conductivity of the epoxy-based composite with the FWCNT-MPCF-Ag hybrid filler increased from 11.2 to 14.9 W/mK by adding Ag nanoparticles of 5 phr. However, the effect of adding the Ag nanoparticles on the thermal conductivity of the epoxy-based composite was much lower than that of the free-standing films; this is associated with the weakened thermal bridge effect for the heat transfer between the carbonaceous fillers due to the lower loading concentration (5 phr) of Ag nanoparticles that possess a spherical shape in polymer composites.

## 4. Conclusions

In this study, carbon-based hybrid films were fabricated using the ultrasonication-assisted vacuum filtration method to design effective heat transfer pathways for 1D-shaped carbon-based fillers in polymer composite systems with hybrid fillers. The bulk density and thermal conductivity of the FWCNT films increased with an increasing ultrasonication time. This FWCNT film bulk density can be associated with the filler loading amount in hybrid filler composite systems, indicating that a high loading amount of fillers is required to achieve the effective formation of the filler-to-filler connection. By using hybrid network systems of nano-sized (FWCNT) and micro-sized (MPCF) carbon materials with a 1D shape, we confirmed the obvious synergistic effect on the thermal conductivity. Moreover, we elucidated that the heat transfer pathway from MPCF to MPCF is the most effective for achieving high thermal conductivity due to the smaller interfacial area and shorter heat transfer path, which can be promoted by longer MPCFs [35]. The FWCNTs in the hybrid network system could act as thermal bridges between neighboring MPCFs, resulting in a synergistic effect on the enhancement of thermal conductivity. It was also confirmed that the intrinsic thermal conductivity of 1D carbon materials is an important factor in determining the thermal conductivity of thermally conductive networks. Furthermore, the thermal interfacial resistance at the intersection points between the thermally conductive carbon materials could be reduced by adding Ag nanoparticles, resulting in higher thermal conductivity. Epoxy-based composites loaded with the FWCNTs, MPCFs, FWCNT-MPCF hybrids, and FWCNT-MPCF-Ag hybrid fillers were also fabricated. A similar trend in thermal conductivity was observed in the polymer matrix composite with carbon-based hybrid films. We expect that the information obtained from this study regarding heat transfer pathways can be used in a wide range of thermal management applications in various electronic devices for heat dissipation.

## Supplementary Materials

The following are available online at https://www.mdpi.com/2073-4360/11/10/1661/s1. Table S1: Summary of the detailed preparation conditions and thermal conductivity values of carbon-based films prepared in this study. Figure S1: Schematic of the thermal conductivity measurement system using the modified laser flash technique. Figure S2: HR-SEM images of the FWCNT-MPCF hybrid films with different MPCF (length: 200 μm) contents. Figure S3: TGA curve of Ag nanoparticles under air atmosphere to 350 °C. Figure S4: Measurement of the Ag nanoparticle sizes using HR-SEM image of the FWCNT-MPCF-Ag hybrid film.
