# Supplementary material for: Effective Heat Transfer Pathways of Thermally Conductive Networks Formed by One-Dimensional Carbon Materials with Different Sizes
Source: Polymers (Basel). 2019 Oct 11;11(10):1661. doi: 10.3390/polym11101661 (PMC6835844; doi:10.3390/polym11101661)
Supplement: Supplementary file 1 [file polymers-11-01661-s001.pdf]

# Effective Heat Transfer Pathways of Thermally Conductive Networks Formed by One-Dimensional Carbon Materials with Different Sizes

Yun Seon Lee[1,2,†], Seung-Yong Lee[1,3,†], Keun Soo Kim[4], Suguru Noda[5], Sang Eun Shim[2,*], Cheol-Min Yang[1,*]

[1] Institute of Advanced Composite Materials, Korea Institute of Science and Technology (KIST), 92 *Chudong-ro*, Wanju-gun, Jeonbuk 55324, Republic of Korea; t14225@kist.re.kr (Y.S.L.); seungyong@lginnotek.com (S.-Y. L.)

[2] Department of Chemical Engineering, Inha University, 100 Inha-ro, Nam-gu, Incheon 22212, Republic of Korea

[3] Magok R&D, LG Innotek, 30 Magokjungang 10-ro, Gangseo-gu, Seoul 07796, Republic of Korea

[4] Department of Physics and Graphene Research Institute, Sejong University, 209 Neungdong-ro, Gwangjin-gu, Seoul 05006, Republic of Korea; kskim2676@sejong.ac.kr

[5] Department of Applied Chemistry, School of Advanced Science and Engineering, Waseda University, 3-4-1 Okubo, Shinjuku-ku, Tokyo 169-8555, Japan; noda@waseda.jp

[*] Correspondence: seshim@inha.ac.kr (S.E.S.); cmyang1119@kist.re.kr (C.-M. Y.)

[†] These authors contributed equally to this work and should be considered co-first authors.

**Table S1.** Summary of the detailed preparation conditions and thermal conductivity values of carbon-based films prepared in this study.

| Sample | FWCNT content (wt%) | MPCF content (wt%) | Ag content (mg) | Sonication time (h) | Heat treatment (1000 ℃) | Thickness (㎛) | Bulk density (kg/m$^3$) | Thermal conductivity (W/mK) |
|---|---|---|---|---|---|---|---|---|
| 1 | 100 | - | - | 0.5 | X | 248 | 210 | 1.65 |
| 2 | 100 | - | - | 2 | X | 228 | 286 | 2.70 |
| 3 | 100 | - | - | 4 | X | 135 | 316 | 4.02 |
| 4 | 100 | - | - | 8 | X | 138 | 360 | 4.71 |
| 5 | 80 | 20 (200 μm) | - | 0.5 | X | 236 | 220 | 2.30 |
| 6 | 60 | 40 (200 μm) | - | 05 | X | 182 | 280 | 3.29 |
| 7 | 40 | 60 (200 μm) | - | 0.5 | X | 166 | 360 | 4.91 |
| 8 | 20 | 80 (200 μm) | - | 0.5 | X | 149 | 350 | 4.63 |
| 9 | 10 | 90 (200 μm) | - | 0.5 | X | 173 | 300 | 4.22 |
| 10 | 5 | 95 (200 μm) | - | 0.5 | X | 179 | 290 | 3.60 |
| 11 | 100 | - | - | 4 | O | 145 | 305 | 4.80 |
| 12 | 100 | - | - | 0.5 | O | 250 | 200 | 2.02 |
| 13 | 80 | 20 (200 μm) | - | 0.5 | O | 229 | 213 | 2.71 |
| 14 | 60 | 40 (200 μm) | - | 0.5 | O | 190 | 272 | 4.02 |
| 15 | 40 | 60 (200 μm) | - | 0.5 | O | 161 | 327 | 5.41 |
| 16 | 20 | 80 (200 μm) | - | 0.5 | O | 150 | 298 | 4.84 |
| 17 | 10 | 90 (200 μm) | - | 0.5 | O | 158 | 289 | 4.25 |
| 18 | 5 | 95 (200 μm) | - | 0.5 | O | 134 | 322 | 3.65 |
| 19 | 40 | 60 (6 mm) | - | 0.5 | X | 185 | 274 | 10.7 |
| 20 | 40 | 60 (6 mm) | 5 | 0.5 | X | 197 | 234 | 14.9 |
| 21 | 40 | 60 (6 mm) | 10 | 0.5 | X | 188 | 333 | 21.0 |
| 22 | 40 | 60 (6 mm) | 15 | 0.5 | X | 200 | 310 | 23.7 |
| 23 | 40 | 60 (6 mm) | 20 | 0.5 | X | 270 | 316 | 25.1 |
| 24 | 40 | 60 (6 mm) | 25 | 0.5 | X | 249 | 343 | 25.8 |

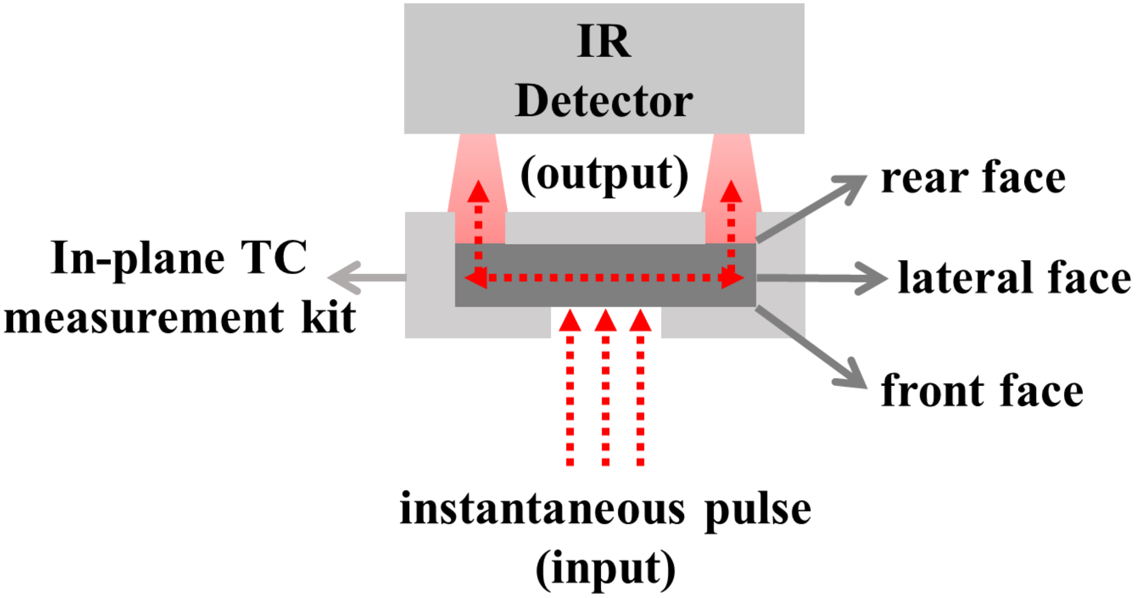


**Figure S1.** Schematic of the thermal conductivity measurement system using the modified laser flash technique.

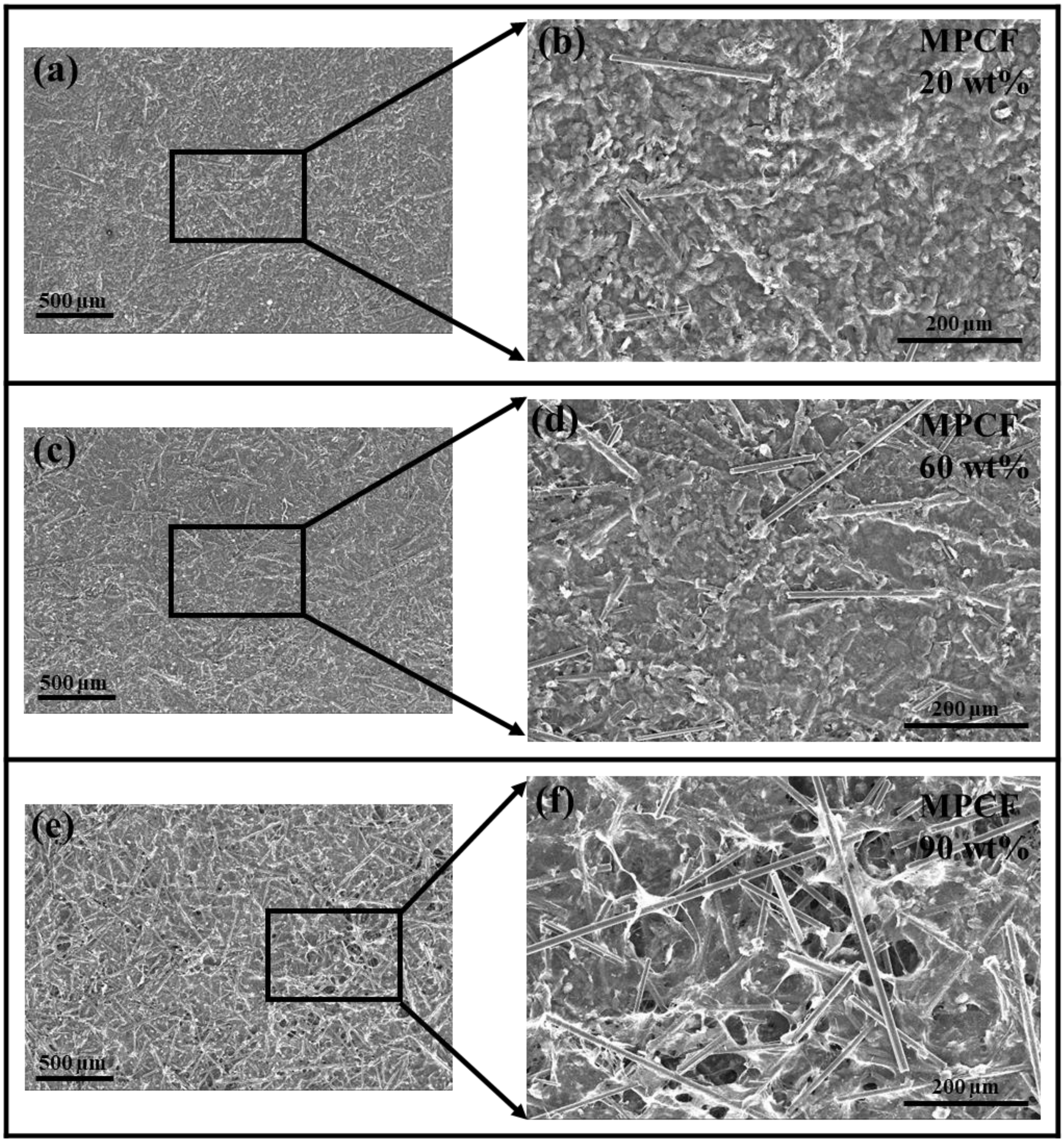


**Figure S2.** HR-SEM images of the FWCNT-MPCF hybrid films with different MPCF (length: 200 μm) contents: (a) and (b) 20 wt%, (c) and (d) 60 wt%, and (e) and (f) 90 wt%. (a), (c), and (e) are low-magnification images, while (b), (d), and (f) are high-magnification images.

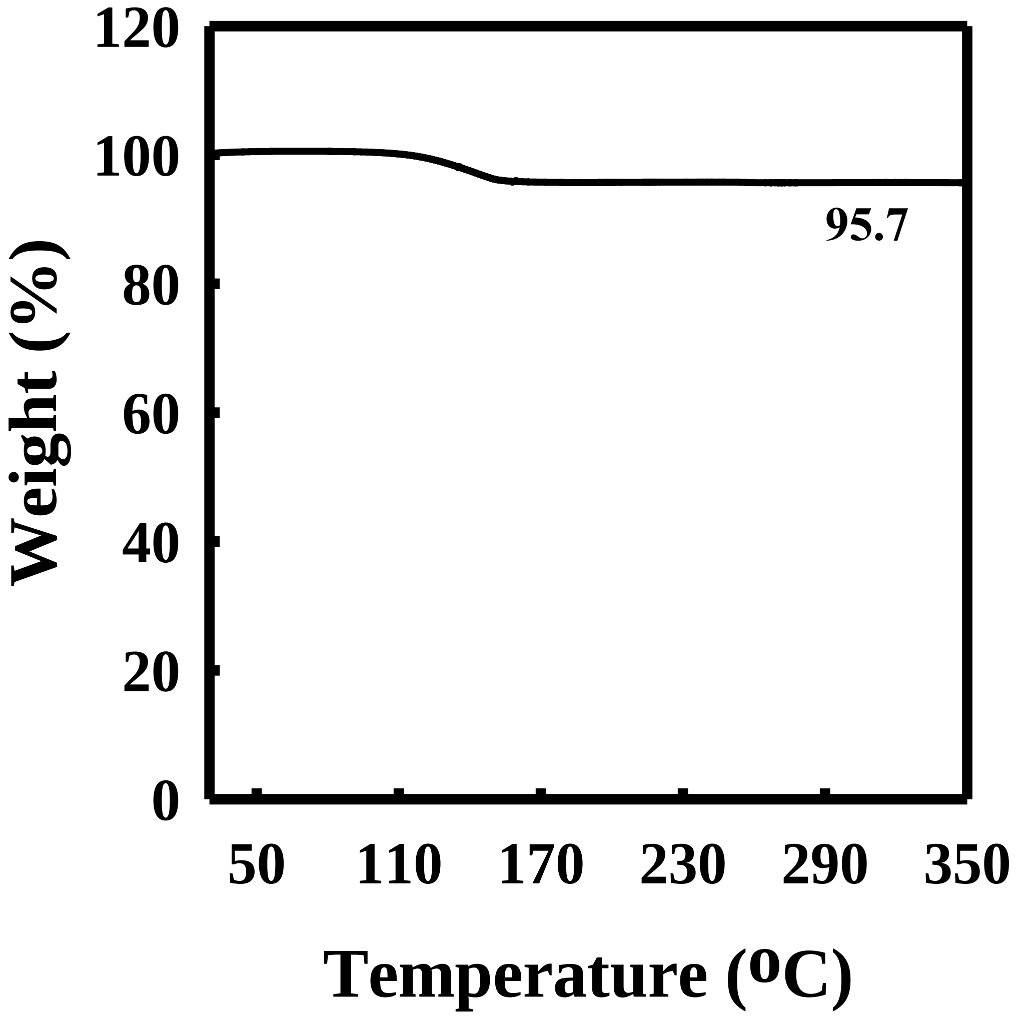


**Figure S3.** TGA curve of Ag nanoparticles under air atmosphere to 350 ºC (heating rate of 10ºC $min^{-1}$).

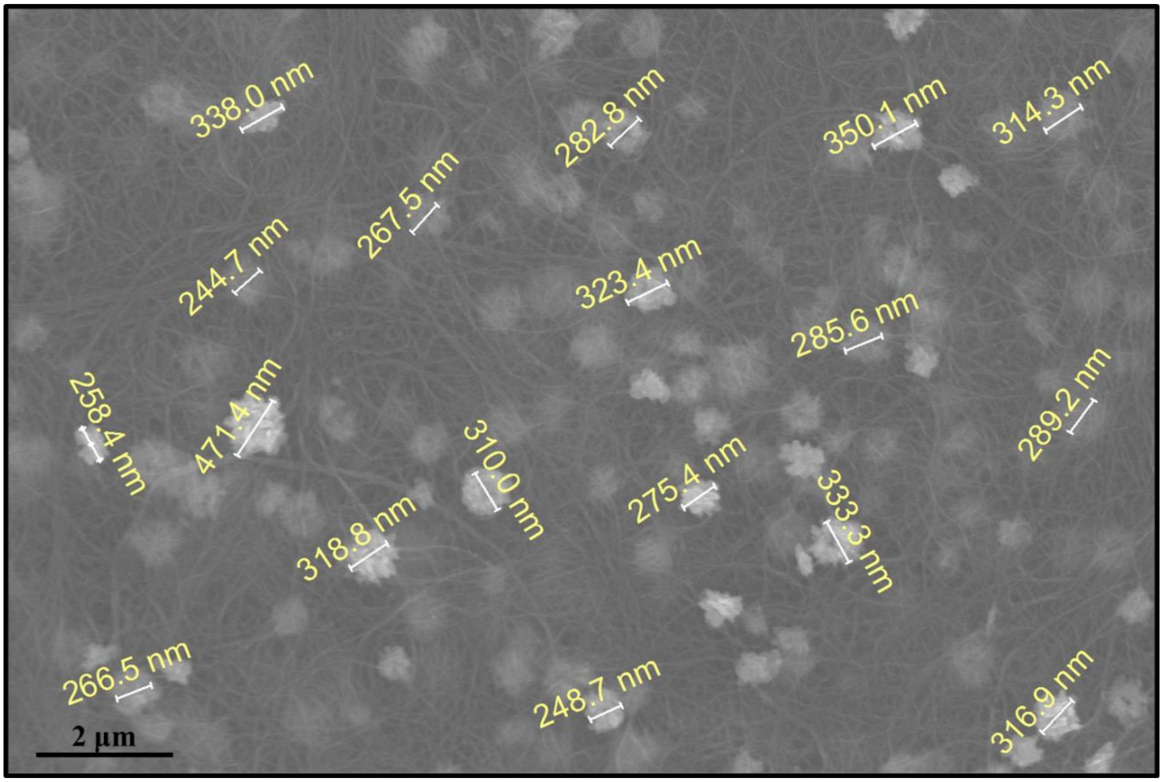


**Figure S4.** Measurement of the Ag nanoparticle sizes using HR-SEM image of the FWCNT-MPCF-Ag hybrid film incorporated with Ag content of 20 mg (MPCF length: 6 mm; MPCF content: 60 wt%; ultrasonication for 0.5 h).
